# Ouabain, a Cardiac Glycoside, Inhibits the Fanconi Anemia/BRCA Pathway Activated by DNA Interstrand Cross-Linking Agents

**DOI:** 10.1371/journal.pone.0075905

**Published:** 2013-10-04

**Authors:** Dong Wha Jun, Mihwa Hwang, Hyun Jung Kim, Soo Kyung Hwang, Sunshin Kim, Chang-Hun Lee

**Affiliations:** 1 New Experimental Therapeutics Branch, Division of Convergence Technology, National Cancer Centre, Goyang, Gyeonggi, Korea; 2 Cancer Cell and Molecular Biology Branch, Division of Cancer Biology, National Cancer Centre, Goyang, Gyeonggi, Korea; University of Hawaii Cancer Center, United States of America

## Abstract

Modulation of the DNA repair pathway is an emerging target for the development of anticancer drugs. DNA interstrand cross-links (ICLs), one of the most severe forms of DNA damage caused by anticancer drugs such as cisplatin and mitomycin C (MMC), activates the Fanconi anemia (FA)/BRCA DNA repair pathway. Inhibition of the FA/BRCA pathway can enhance the cytotoxic effects of ICL-inducing anticancer drugs and can reduce anticancer drug resistance. To find FA/BRCA pathway inhibitory small molecules, we established a cell-based high-content screening method for quantitating the activation of the FA/BRCA pathway by measuring FANCD2 foci on DNA lesions and then applied our method to chemical screening. Using commercial LOPAC1280 chemical library screening, ouabain was identified as a competent FA/BRCA pathway inhibitory compound. Ouabain, a member of the cardiac glycoside family, binds to and inhibits Na^+^/K^+^-ATPase and has been used to treat heart disease for many years. We observed that ouabain, as well as other cardiac glycoside family members―digitoxin and digoxin―down-regulated FANCD2 and FANCI mRNA levels, reduced monoubiquitination of FANCD2, inhibited FANCD2 foci formation on DNA lesions, and abrogated cell cycle arrest induced by MMC treatment. These inhibitory activities of ouabain required p38 MAPK and were independent of cellular Ca^2+^ ion increase or the drug uptake-inhibition effect of ouabain. Furthermore, we found that ouabain potentiated the cytotoxic effects of MMC in tumor cells. Taken together, we identified an additional effect of ouabain as a FA/BRCA pathway-inhibiting chemosensitization compound. The results of this study suggest that ouabain may serve as a chemosensitizer to ICL-inducing anticancer drugs.

## Introduction

Most cancer therapies, including conventional chemotherapy and radiotherapy, are based on DNA damage-induced tumor cell death. These cancer therapeutic DNA-damaging agents activate cellular responses, such as cell cycle arrest, DNA repair, and cell death, which are collectively called the DNA damage response. When DNA damage occurs in tumor cells, apoptosis is activated to remove cells harboring severe DNA damage, whereas cell survival processes, such as cell cycle arrest and DNA repair, are activated to recover from DNA lesions and for survival. Activation of the DNA repair process against DNA damaging agents in tumor cells decreases the efficacy of anticancer drugs and eventually leads to drug resistance. Combinations of conventional cancer therapy with inhibition of the DNA repair process are expected to enhance therapeutic efficacy. Thus, DNA repair pathways in cancer cells are regarded as promising targets for anticancer drug development [1-4].

Eukaryotic cells activate DNA damage repair pathways depending on the type of DNA damage incurred. Among the DNA damage repair pathways, the Fanconi anemia (FA)/BRCA pathway is required for the repair of DNA interstrand cross-links (ICLs), one of the most severe forms of DNA damage caused by anticancer agents such as cisplatin and mitomycin C (MMC). DNA ICL-inducing agents have been used to treat cancers, such as multiple myeloma, testicular, ovarian, bladder, head and neck, and breast cancers [5-7]. During chemotherapy, activation of the FA/BRCA pathway might lead to treatment failure. The FA/BRCA pathway was reported to be responsible for resistance to cisplatin treatment in ovarian tumors [8]. Also, several publications have reported that the FA/BRCA pathway confers resistance to DNA ICL-inducing agents in many cancers, such as glioma and multiple myeloma [9,10]. Thus, inhibition of the FA/BRCA pathway may make resistant tumor cells more sensitive to ICL-inducing agents. Recently, cell-based and cell-free screening methods have been adopted to identify FA/BRCA pathway inhibitors. Several chemicals, including curcumin and a menadione analog, have been shown to sensitize tumor cell lines to cisplatin treatment [11,12].

The FA/BRCA pathway orchestrates FA proteins to repair DNA ICL lesions. In response to DNA ICLs, the FA nuclear core complex composed of at least 8 FA proteins (FANCA, -B, -C, -E, -F, -G, -L, and -M) monoubiquitinates the FANCD2 and FANCI proteins. Monoubiquitinated FANCD2 and FANCI proteins are recruited to the DNA ICL lesion and subsequently interacts with DNA repair-related proteins, such as RAD51 and BRCA1, to repair DNA damage [13,14]. Accumulation of monoubiquitinated FANCD2 on DNA lesions is visualized as nuclear foci using immune-staining with a FANCD2-specific antibody under a fluorescence microscope. The monoubiquitinated FANCD2 foci are used as markers for DNA ICL damage, which could be used for massive screening when combined with high-content screening tools [11,15]. In this study, we performed chemical library screening to find a small molecule inhibitor of the FA/BRCA pathway adopting a cell-based, high-content screening system using monoubiquitinated FANCD2 foci as markers for the activation of the FA/BRCA pathway. We identified ouabain [1β,3β,5β,11α,14,19-hexahydroxycard-20(22)-enolide 3-(6-deoxy-α-L-mannopyranoside)], a well-known cardiac glycoside that binds and inhibits Na^+^/K^+^-ATPase, as a novel inhibitor of FA/BRCA pathway activation.

## Materials and Methods

### Cell lines and cell culture

U2OS osteosarcoma cells were acquired from KCLB (Seoul, Korea) and cultured in Dulbecco’s modified Eagle’s medium (DMEM) supplemented with 10% fetal bovine serum (FBS; Hyclone, Logan, UT) and 2 mM glutamine at 37°C in a 5% CO_2_ atmosphere.

### Chemicals and antibodies

MMC, curcumin, ouabain, digitoxin, digoxin, thapsigargin, and nifedifin were purchased from Sigma-Aldrich Co. (St. Louis, MO). Cisplatin and MAPK kinase inhibitors (PD 98059, SB203580, and SP600125) were obtained from Calbiochem (San Diego, CA). Antibodies against FANCD2 (NB 100-182) and FANDI (A300-212A) were purchased from NOVUS (Littleton, CO) and Bethyl Laboratories (Montgomery, TX), respectively. Anti-MAPK family (#9926) and phospho-MAPK family (#9910) molecules were acquired from Cell Signaling Technology (Danvers, MA). Anti-PARP (556494) was purchased from BD Pharmingen (San Jose, CA). All other chemicals and reagents were purchased from Sigma-Aldrich Co. unless otherwise specified.

### Immunofluorescence

One day before treatment, cells were seeded at 0.5 × 10^4^/well in black 96-well plates with clear, flat bottoms (Costar, Corning, NY). After treatment, cells were rinsed with phosphate-buffered saline (PBS), fixed with 3.7% formaldehyde in PBS for 20 min at room temperature, and permeabilized with 0.2% Triton X-100 in PBS for 5 min on ice. Nonspecific binding was blocked by incubating cells with 1% bovine serum albumin and 0.02% Triton X-100 in PBS for 30 min at room temperature. The cells were sequentially incubated with anti-FANCD2 antibody for 3 h at room temperature, Alexa Fluor 488-conjugated anti-rabbit IgG antibody (1:500; Molecular Probes, Carlsbad, CA) for 1 h, and Hoechst 33342 (10 μg/ml; Molecular Probes) for 10 min. The cells were washed three times with PBS for 10 min each time and were visualized using an In Cell Analyzer 1000 (GE Healthcare, Buckinghamshire, UK).

### Acquisition and analysis of images

The total count and area of FANCD2 foci were measured using IN Cell Analyzer 1000. Images of cells after immunofluorescence were acquired with the automated fluorescence microscope platform of the IN Cell Analyzer using a 20× objective lens. Images from more than five fields per well were collected to obtain data from 400 to 600 cells. The filter sets used for detection of Hoechst 33342 and Alexa Fluor 488 signals were D360/40 (excitation)-HQ535/50 (emission) and D475/20 (excitation)-HQ 535/20 (emission), respectively. The acquired images were analyzed using the Multi Target Analysis (MTA) module of the IN Cell Analyzer 1000 Workstation software (v3.4) as previously described [16,17]. MTA module measured fluorescence intensity and area (µm^2^) of nuclear (based on Hoechst 33342 staining) and targeted organells (based on Alexa Fluor 488 staining) respectively. The fluorescence area of Alexa Fluor 488 represented FANCD2 foci. Previously we have reported that foci total area per cell exhibited good correlation with extent of DNA damage [16].

### Western blotting

For FANCD2 and FANCI detection, cell lysates were prepared by suspending 4 × 10^5^ cells in 100 µl of NuPAGE LDS sample buffer containing 5% mercaptoethanol (Invitrogen, Carlsbad, CA). The cell lysates were kept on ice for 10 min and boiled at 95°C for 5 min. Equal amounts of lysate proteins were separated on 3–8% NuPAGE Tris-acetate gels and transferred to nitrocellulose membranes using the iBlot Gel Transfer System (Invitrogen). After blocking with 5% skim milk in TBST [20 mM Tris (pH 7.5), 135 mM NaCl, and 0.05% Tween 20], the membranes were incubated with the indicated antibodies. The membranes were washed and then incubated with horseradish peroxidase-conjugated anti-rabbit IgG (1:5000; Cell Signaling Technology). After extensive washing, the proteins were visualized by chemiluminescence using the ECL reagent (GE Healthcare). For MAPK family detection, whole-cell lysates were prepared by suspending 2 × 10^6^ cells in 100 µl of 1 x SDS sample buffer [62.5 mM Tris (pH 6.8), 2% SDS, 10% glycerol, 50 mM DTT, 0.01% bromophenol blue]. The cell lysates were sonicated for 10 sec, boiled for 5min and cleared by centrifugation at 15,000 × g for 10 min at 4°C. Equal amounts of lysate protein were separated on a 4–12% gradient or 15% Tris-Glycine PAGE and transferred to Immobilon P membranes (Millipore, Billerica, MA). Immunoblotting steps were done as described above.

### Cell cycle analysis

Cells treated with MMC and chemical compounds were washed with PBS, fixed with cold 70% ethanol, and stored at -20°C for 16 h or longer. The fixed cells were washed with PBS and then incubated in DNA staining solution (10 μg/ml propidium iodide + 1 µg/ml RNase A; Sigma-Aldrich Co.) for 20 min at room temperature in the dark. After incubation, the samples were analyzed by using FACSCalibur (BD Pharmingen, San Jose, CA).

### Cell survival assay

Cells were seeded at 2 × 10^4^ per well in a 96-well plate and treated with MMC and chemical compounds at the indicated concentrations. After incubation for the indicated time, cells were washed with PBS and 100 µl of 3-(4,5-dimethylthiazol-2-yl)-2,5-diphenyl tetrazolium bromide (MTT) was added at a concentration of 0.5 mg/ml. After incubation in a CO_2_ incubator at 37°C for 2 h, insoluble crystals were completely dissolved in dimethyl sulfoxide. The absorbance at 540 nm was measured using a Versamax microplate reader (Molecular Devices, Sunnyvale, CA).

### Chemical library screening

The LOPAC1280 chemical library was purchased from Sigma-Aldrich Co (St. Louis, MO). One hour before treatment with MMC (200 ng/ml), each of the 1280 compounds in the chemical library was added to cells grown on black 96-well plates with clear, flat bottoms (Costar) at a final concentration of 5 µM. After 24 h incubation, cells were immunofluorescence-stained and then analyzed using the IN Cell Analyzer. We calculated the means and standard deviation (SD) of FANCD2 foci count from four positive control wells (only MMC-treated) of each plate and selected compounds that showed FANCD2 foci count reduction by more than 3 × SD compared with the positive control.

### Statistical analysis

Data are presented as mean values ± standard error of the mean (SEM). All statistical analyses were performed with GraphPad Prism version 5.03 (GraphPad Software, San Diego, CA). Comparisons between two groups were carried out using Student’s t-test for unpaired data. Differences between groups were considered statistically significant at *P < 0.05*. Combination Index (CI) was calculated using CalcuSyn software (Biosoft, Cambridge) based on the multiple drug-effect equation of Chou-Talalay.

## Results

### Ouabain was identified as having FA/BRCA pathway-inhibiting activity

**Figure 1:**
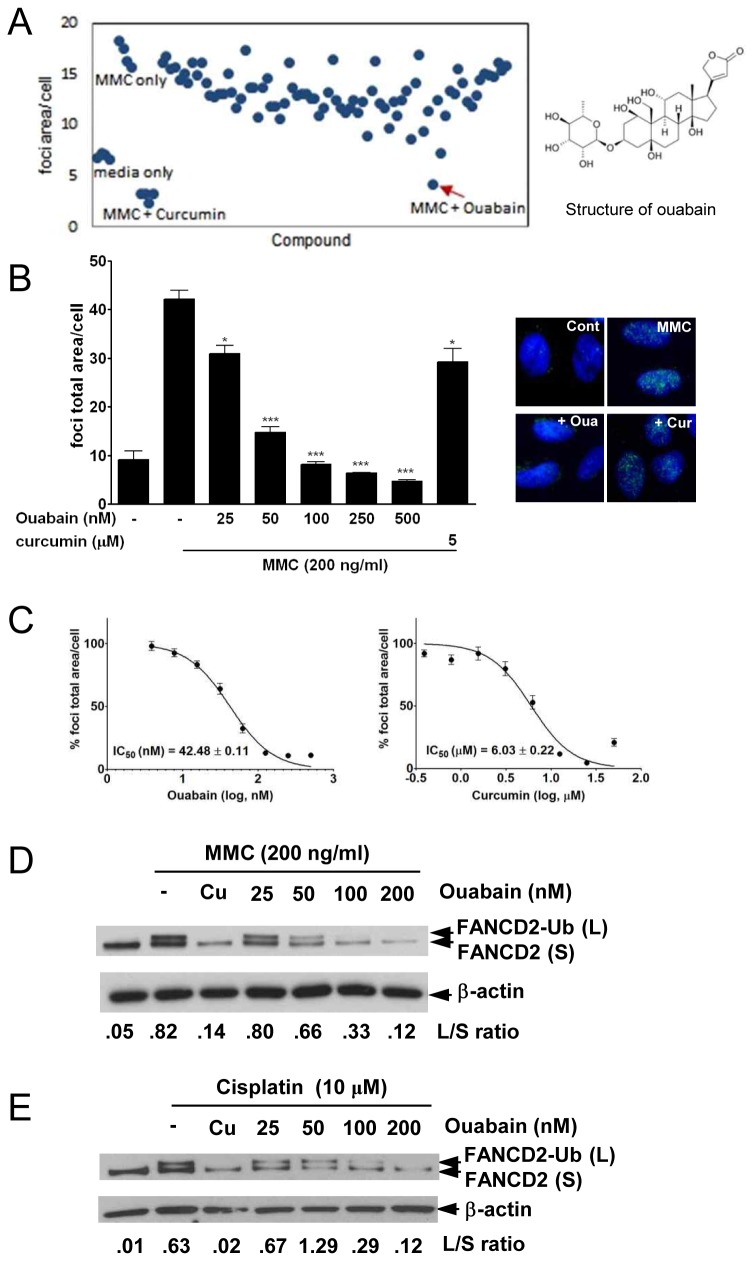
Ouabain inhibits the FA/BRCA pathway. (A) The data from chemical library screening and chemical structure of ouabain. U2OS cells were pre-treated with 5 µM of each compound for 1 h prior to incubation in 200 ng/ml MMC for 24 h. Representative data from plate containing ouabain compound are shown. Each dot in the scattergram represents FANCD2 foci area per cell after treatment with each compound tested. (B) Ouabain inhibits MMC-induced FANCD2 foci formation. U2OS cells were pretreated with the indicated concentration of ouabain or curcumin for 1 h and then treated with 200 ng/ml MMC for 24 h. After incubation, the cells were fixed and processed for FANCD2 immunofluorescence and the FANCD2 foci were analyzed with an IN Cell Analyzer. Representative graphs and images from three independent experiments are shown. Values represent the means ± SEM (Student’s *t*-test, ***, *P* < 0.001; *, *P* < 0.05). (C) U2OS cells treated as described in (B) were analyzed with an IN Cell Analyzer. % foci total area/ cell represents relative % to MMC-only treated samples. IC_50_ was calculated using GraphPad Prism version 5.03. Graphs and values represent the means ± SEM from three independent experiments. (D) Ouabain inhibits MMC-induced FANCD2 monoubiquitination. Protein extracts fromU2OS cells treated as described in (B) were analyzed by Western blotting using antibodies against FANCD2 and β-actin. Values shown represent the ratio of FANCD2 (L)/FANCD2 (S). (E) Ouabain inhibits FANCD2 monoubiquitination after cisplatin treatment. Western blotting was performed with protein extracts from U2OS cells treated with 10 µM cisplatin.

To identify FA/BRCA pathway inhibitors, we established a cell-based high-content screening method measuring FANCD2 foci as a marker for FA/BRCA pathway activation after immunofluorescence staining. MMC was used as an ICL inducer to activate the FA/BRCA pathway, and curcumin was used as a control of competent FA/BRCA inhibitory chemicals [11]. For screening quality control, each plate contained four media-only wells, four MMC-only-treated wells, and four MMC + curcumin-treated wells (Figure 1A). To confirm the significance of the screen, we calculated the Z’ factor for the assay, defined as 1 – [3(SD of the MMC-only controls + SD of the media-only controls)/(mean of the MMC-only controls – mean of the media-only controls)]. The Z’ factor of the assay was 0.882, where a Z’ factor of 1 is ideal and a Z’ factor greater than 0.5 indicates excellent screening quality [18]. We screened a LOPAC1280 chemical library using this method and found that ouabain was a competent FA/BRCA pathway inhibitor (Figure 1A and Table S1). In Figure 1B, we confirmed that ouabain could inhibit FANCD2 foci formation in a dose dependent manner. In the presence of 500 nM and 25 nM ouabain, the FANCD2 foci area per cell declined from 42.19 ± 3.2 to 6.43 ± 0.20 and 30.97 ± 2.96, respectively. FANCD2 foci decreased from 42.19 ± 3.2 to 29.25 ± 4.79 by treatment with 5 µM curcumin, a similar degree to the 25 nM of ouabain treatment, showing that ouabain has more potent inhibitory activity than curcumin, which is a well-known FA/BRCA pathway inhibitor. IC_50_ values of ouabain and curcumin in FA/BRCA pathway inhibition were 42.48 ± 0.11 nM and 6.03 ± 0.22 µM respectively. Inhibitory activity of ouabain was 142-fold higher than curcumin (Figure 1C).

We conducted Western blotting to check the activation status of FANCD2 protein after MMC treatment (Figure 1D). The data showed that ouabain treatment decreased FANCD2 protein levels as curcumin treatment did. We examined FANCD2 mRNA levels by real time RT-PCR and found that level of FANCD2 mRNA was lower in ouabain treated cells (Figure S1). The reduction in levels of FANCD2 and FANCI protein following treatment with ouabain and curcumin could not be rescued by treatment with the proteasome inhibitor MG132 (Figure S1). These results indicate that reduced FANCD2 protein expression after ouabain treatment might result from transcriptional repression but not from enhanced proteasomal degradation. To check the possibility that ouabain affects the transcription of other DNA damage regulators, we performed real time RT-PCR (Figure S2). Ouabain repressed the transcription of FANCF and ERCC4. However, mRNA levels of FANCA, Rad51, XRCC5 and XRCC6 did not change after ouabain treatment.

We also observed that monoubiquitination of FANCD2 was inhibited by ouabain in a dose-dependent manner (Figure 1D). Ouabain also inhibited monoubiquitination of FANCD2 induced by cisplatin, another ICL inducer (Figure 1E). In addition, we observed FA/BRCA pathway inhibition by ouabain and cardiac glycoside family in different cell lines such as HeLa cervical cell line (Figure S3) and CWR22 prostate cancer cell line (data not shown). From these results, we confirmed that ouabain could affect FA/BRCA pathway activation through repression of FANCD2 expression and through inhibition of FANCD2 monoubiquitination upon ICL induction.

### Other glycoside family chemicals also exhibited FA/BRCA inhibitory activity

**Figure 2:**
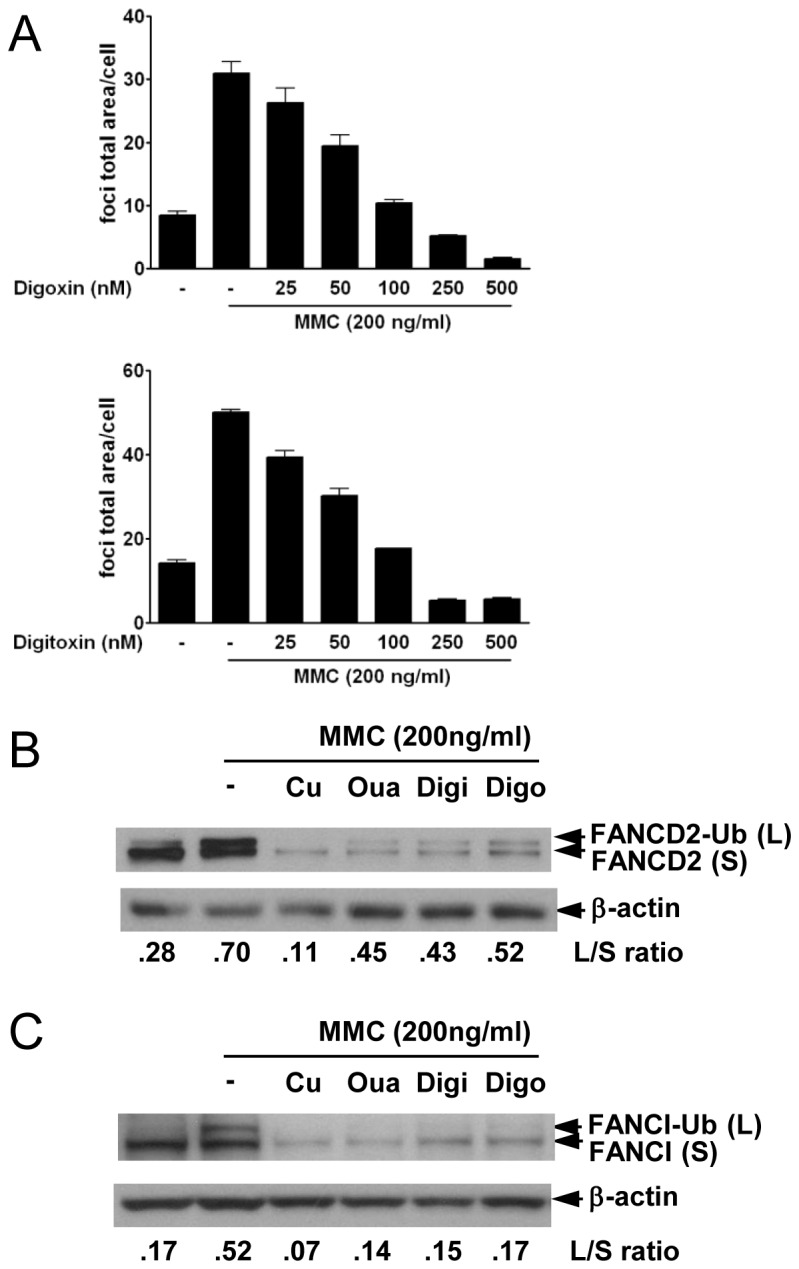
Cardiac glycoside family members inhibit the FA/BRCA pathway. (A) U2OS cells were pretreated with the indicated concentration of digitoxin or digoxin for 1 h and then incubated in 200 ng/ml MMC for 24 h. After incubation, the cells were fixed and processed for FANCD2 immunofluorescence and the FANCD2 foci were analyzed with IN Cell Analyzer. Representative graphs from three independent experiments are shown. Values represent the means ± SEM.(B) (C) Protein extracts from U2OS cells pretreated with 5 µM curcumin, 100 nM ouabain, 100 nM digitoxin and 100 nM digoxin and incubated in 200 ng/ml MMC for 24 h as described in (A) were analyzed by Western blotting using antibodies against (B) FANCD2, (C) FANCI, and β-actin. L/S values shown represent the ratio of FANCD2 (L)/FANCD2 (S) or FANCI (L)/FANCI (S).

Ouabain is a member of the cardiac glycoside family, which has been used to treat heart failure. Cardiac glycosides are naturally derived compounds that bind to and inhibit Na^+^/K^+^ ATPase [19,20]. Na^+^/K^+^ ATPase is a ubiquitous membrane protein that carries out active transport of K^+^ ion into cells and Na^+^ ions out of cells by ATP hydrolysis, maintaining electrolyte and fluid balance in cells. Because the cardiac glycoside family shares a common structural motif, we examined whether other cardiac glycosides (i.e., digoxin and digitoxin) could inhibit the FA/BRCA pathway. In Figure 2A, FANCD2 foci induced by MMC were reduced by treatment with digoxin and digitoxin in a dose-dependent manner. Western blot data also showed that digoxin and digitoxin decreased FANCD2 expression levels and inhibited monoubiquitination of FANCD2 (Figure 2B). Because ICLs activate FA core complex proteins to monoubiquitinate FANCI, as well as FANCD2, and the monoubiquitinated FANCD2-FANCI heterodimer relocalizes to ICLs [21,22], we tested whether FANCI is also affected by treatment with cardiac glycosides. In Figure 2C, Western blot data showed that total expression levels and monoubiquitination of FANCI decreased by treatment with cardiac glycosides (ouabain, digoxin, and digitoxin) and curcumin. Reduced FANCI protein level after ouabain treatment might result from transcriptional repression but not from proteasomal degradation (Figure S1). These data indicated that cardiac glycosides, including ouabain, digoxin, and digitoxin, could inhibit activation of the FA/BRCA pathway.

### Ouabain disturbs MMC-induced cell cycle arrest at the S phase

**Figure 3:**
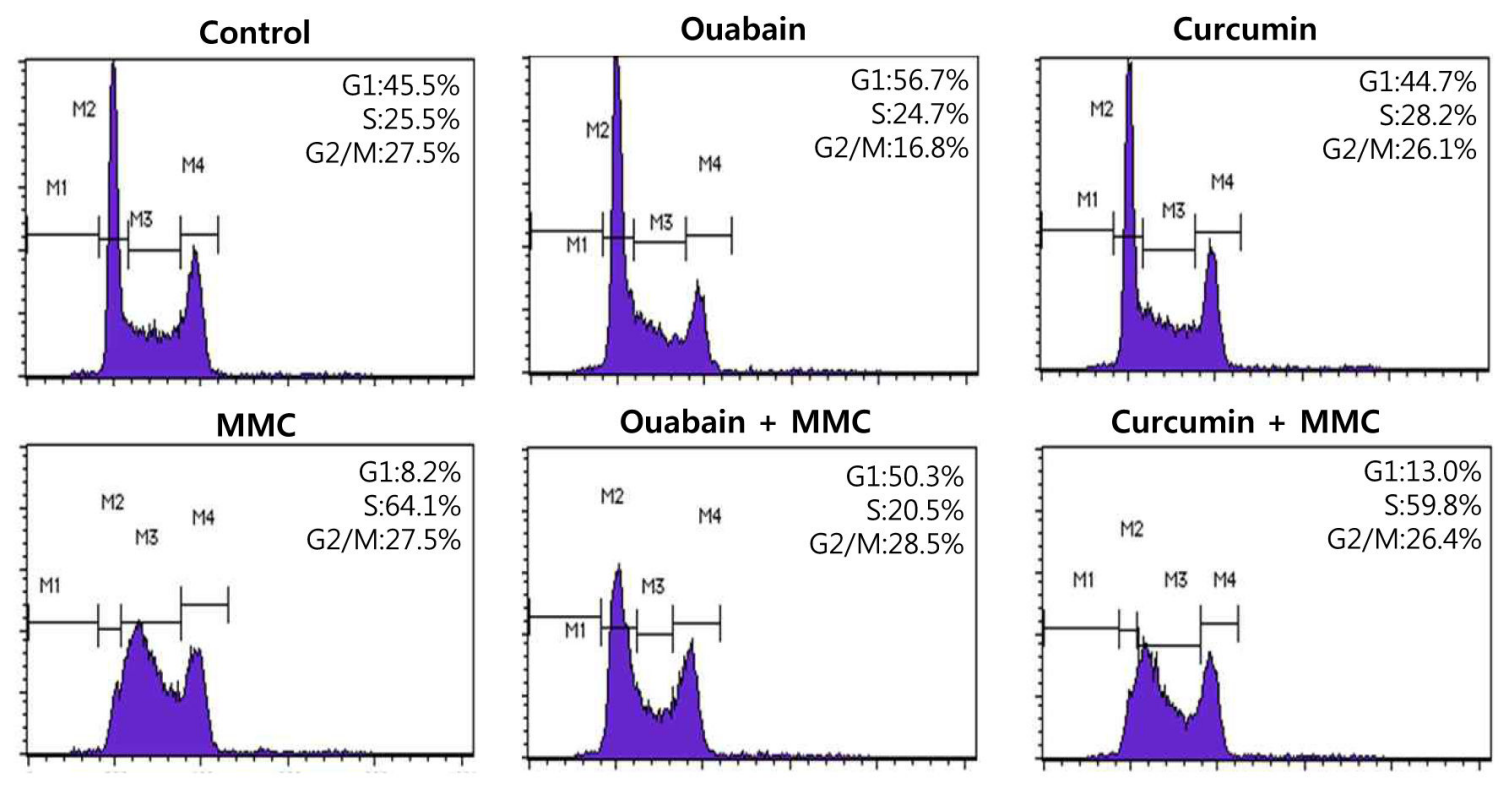
Ouabain disturbed MMC-induced S phase arrest. U2OS cells were pretreated with 50 nM ouabain or 5 µM curcumin for 1 h and then incubated with 200 ng/ml MMC for 24 h. After incubation, the cells were fixed, stained with propidium iodide, and subjected to flow cytometric DNA content analysis. Representative DNA content profiles from three independent experiments are shown.

Previously, ouabain was reported to induce cell cycle arrest in G2/M phase when used to treat thyroid cancer cell lines or neuroblastoma cells [23,24]. Since FANCD2 monoubiquitination mainly occurs in the S phase, cell cycle arrest at the G2/M phase might decrease FANCD2 monoubiquitination. Therefore, we examined whether ouabain could induce cell cycle arrest at the G2/M phase in U2OS cells. Flow cytometric analysis indicated that 50 nM of ouabain did not affect cell cycle progression of U2OS cells (Figure 3, upper panels). However, ouabain inhibited MMC-induced S phase arrest, whereas curcumin did not affect MMC-induced cell cycle arrest (Figure 3, lower panels). These results demonstrated that ouabain did not affect cell cycle progression directly in U2OS cells but inhibited MMC-induced S phase arrest.

### Ouabain inhibits the FA/BRCA pathway through p38 MAPK kinase activation

We further clarified the FA/BRCA pathway inhibitory mechanism of ouabain. First we examined whether intracellular Ca^2+^ ion concentration fluctuation is involved in the inhibition of the FA/BRCA pathway by ouabain because ouabain was reported to induce Ca^2+^ ion oscillations at low doses (sub-100 nM) [25]. When we analyzed the intracellular Ca^2+^ ion concentration by the fluo-4/AM staining method, we found that ouabain treatment negligibly increased the intracellular Ca^2+^ ion concentration compared to thapsigargin treatment (Figure S4). In addition, nifedifin, a Ca^2+^ ion channel blocker, could not recover FA/BRCA pathway inhibition induced by ouabain (Figure S5). Taken together, FA/BRCA pathway inhibition by ouabain was independent of the intracellular Ca^2+^ ion concentration fluctuation.

Since ouabain was also reported to induce drug resistance through stimulation of MDR1 gene expression [26], we tested whether ouabain inhibits the FA/BRCA pathway by blocking accumulation of MMC inside cells. Usually cells were pretreated with ouabain 1 h before MMC treatment and then incubated for 24 h in medium containing ouabain+MMC. To remove the influence of the inhibition of MMC accumulation by ouabain, we first added MMC for 6 h, changed the medium to remove MMC, and then incubated in MMC-free media in the presence or absence of ouabain for 18 h. Posttreated ouabain inhibited MMC-induced FANCD2 foci formation to a similar extent as pretreated ouabain did (Figure S6). These results indicate that FA/BRCA pathway inhibition by ouabain was not due to blocked MMC accumulation.

**Figure 4:**
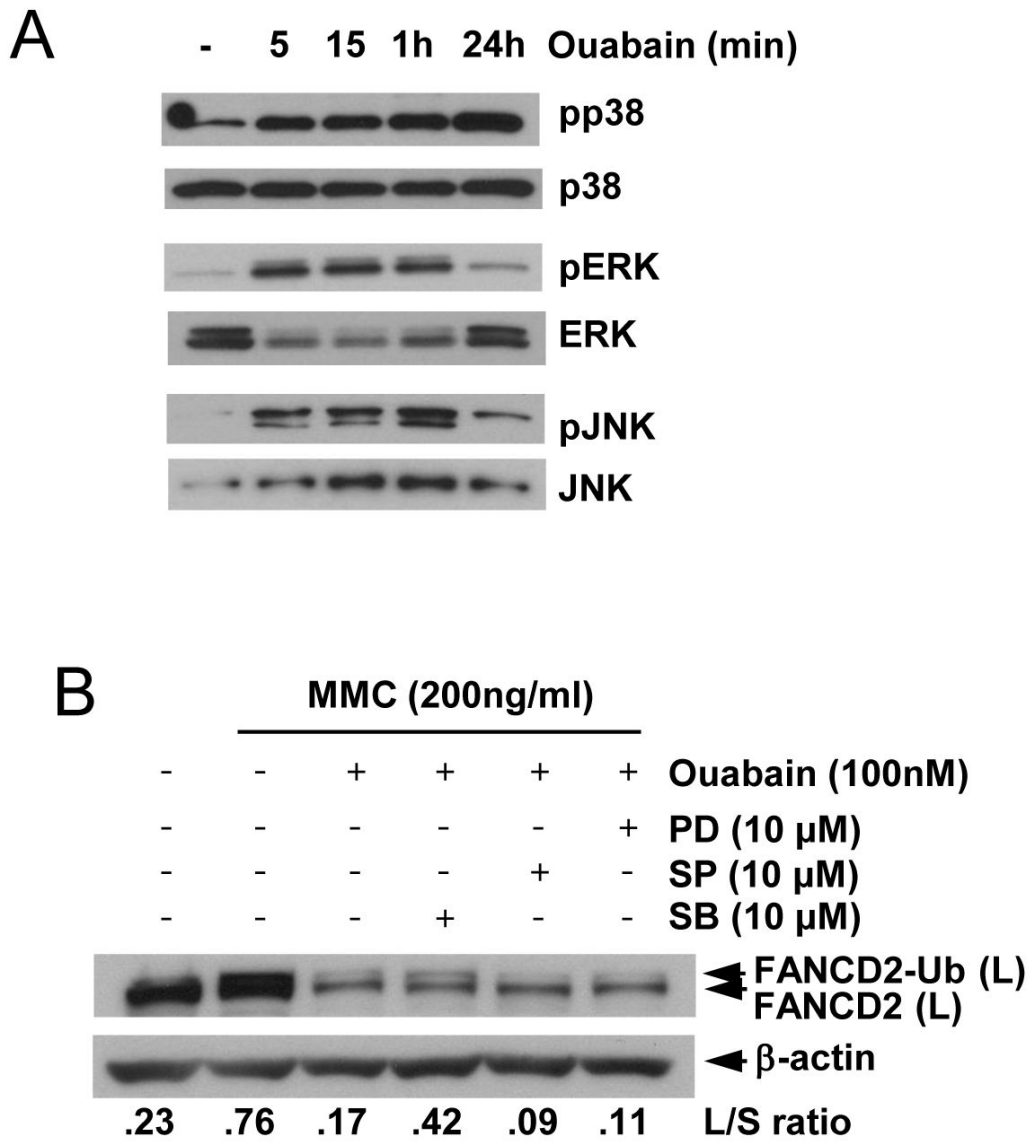
Ouabain inhibits the FA/BRCA pathway through p38 kinase. (A) Protein extracts from U2OS cells treated with 100 nM ouabain for the indicated times were analyzed by Western blotting using antibodies against phosphorylated p38, p38, phosphorylated ERK, ERK, phosphorylated JNK, and JNK. (B) U2OS cells were pretreated with the indicated concentration of MAPK inhibitors for 30 min and then with 100 nM ouabain for 30 min and incubated in 200 ng/ml MMC for 24 h. The cell lysates were subjected to Western blot analysis using antibodies to FANCD2 and β-actin. PD, PD 98059 (ERK inhibitor); SP, SP 600215 (JNK inhibitor); SB, SB 203580 (p38 inhibitor).

Next we checked whether MAP kinase activation is involved in FA/BRCA pathway inhibition by ouabain because Na^+^/K^+^ ATPase, to which ouabain binds, can transduce signals through the SRC-Ras-Raf-MAPK cascade [27]. Western blot data showed that ouabain induced phosphorylation of JNK, ERK, and p38 in the U2OS cell line as early as 5 min after treatment (Figure 4A). The phosphorylation of p38 was sustained until 24 h after ouabain treatment, whereas JNK and ERK phosphorylation levels decreased to background levels. To identify which kinase is involved in FA/BRCA pathway inhibition by ouabain, we added the MAPK inhibitors SB203580 (p38 inhibitor), SP600125 (JNK inhibitor), and PD 98059 (MEK inhibitor, upstream of ERK) before the ouabain+MMC treatment. Western blot data showed that SB203580 (p38 inhibitor) prevented FANCD2 monoubiquitination inhibition by ouabain (Figure 4B). These data correlated with prolonged phosphorylation of p38 up to 24 h after ouabain treatment (Figure 4A). These results indicate that p38 kinase is involved in FA/BRCA pathway inhibition by ouabain.

### Ouabain enhanced the cytotoxicity of MMC

**Figure 5:**
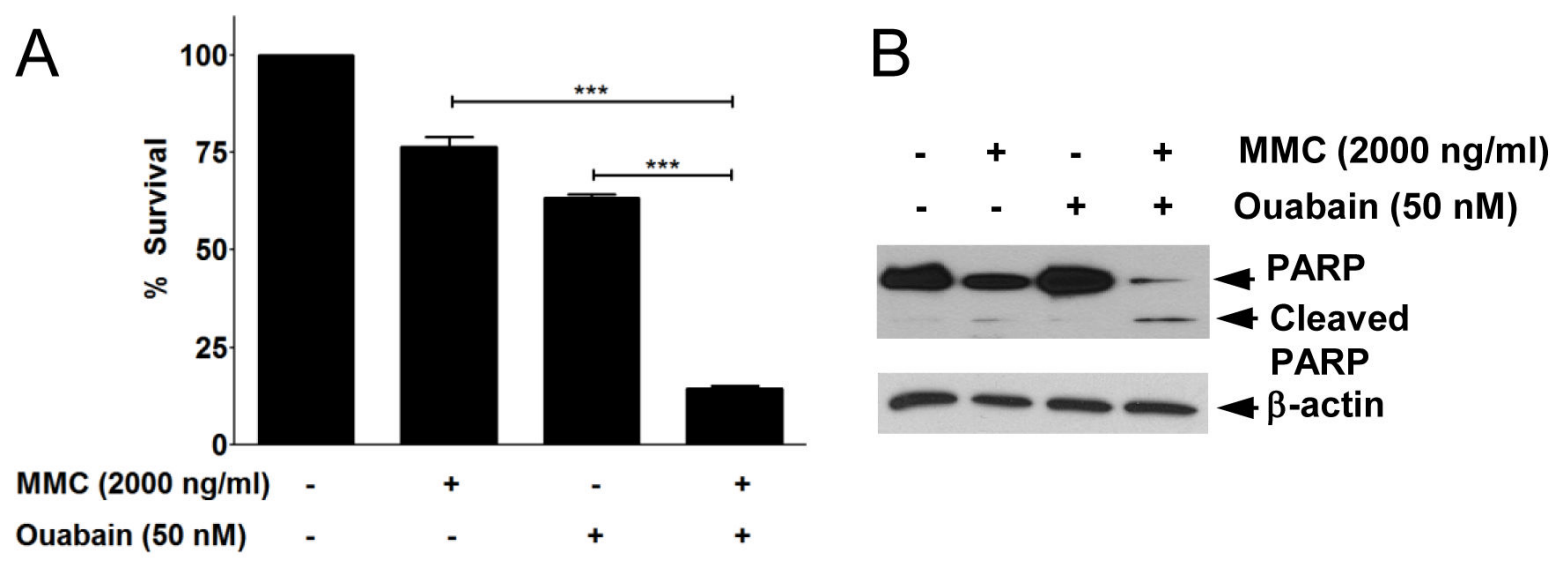
Ouabain enhances cytotoxicity of MMC against U2OS cells. (A) U2OS cells were pretreated with the 50 nM ouabain for 1 h and then treated with 2000 ng/ml MMC. After incubation for 48 h, cell viability was measured with the MTT assay. Values represent the means ± SEM from three independent experiments (Student’s *t*-test, ***, *P* < 0.001). (B) Protein extracts from U2OS cells treated as described in (A) were analyzed by Western blotting using antibodies against PARP-1. Proform of PARP (116 kDa) and cleaved PARP (85 kDa) are indicated.

Because several reports have suggested that co-treatment with DNA repair inhibitors enhances the efficacy of anticancer drugs, we examined whether ouabain, as a FA/BRCA pathway inhibitor, could potentiate cytotoxicity of MMC against tumor cells. We checked cell survival 48 h after incubation with MMC in the presence and absence of ouabain. MTT assay indicated that groups treated only with MMC or ouabain showed 76.34 ± 4.37% or 63.25 ± 1.78% survival, respectively (Figure 5A). In contrast, combined treatment of MMC and ouabain decreased % survival to 14.41 ± 1.06%. We calculated Combination Index (CI) using CalcuSyn software which is based on the multiple drug-effect equation of Chou-Talalay [28]. In this analysis, CI value < 0.9 implies synergism, CI = 0.9-1.1 implies additive and CI > 1.1 indicates antagonism, respectively. CI values at effective doses of ED50, ED70 and ED90 were 0.982, 0.669 and 0.457 respectively, suggesting that ouabain and MMC have synergistic effects at this concentration range. To investigate whether cell death induced by combined treatment of ouabain and MMC was due to apoptosis, we checked PARP cleavage, which is a marker of apoptosis. The level of cleaved PARP in cells co-treated with MMC and ouabain was higher than those in cells treated only with MMC or ouabain (Figure 5B). These results indicate that ouabain could significantly sensitize U2OS cells to MMC treatment.

## Discussion

The DNA repair process is an emerging target in anticancer drug development. Modulation of the DNA repair process can be approached using two strategies in the cancer therapeutic field. The first is a combination therapy with DNA repair inhibitors co-treated as a sensitizer to enhance conventional radio- or chemotherapeutic efficacy. The second is a monotherapy with DNA repair inhibitors that can selectively kill tumor cells with deficiency in other DNA repair pathways by inducing synthetic lethality [1].

FA/BRCA pathway inhibitors can be used both in combination therapy and monotherapy, and is thus a promising target in anticancer drug development. In the case of combination therapy, inhibition of the FA/BRCA pathway could lead to sensitization to ICL-inducing agents because the FA/BRCA pathway plays a role in cellular response to ICL-inducing agents. With regard to the application in monotherapy, FA/BRCA pathway deficiency shows synthetic lethality with inhibition of ATM, which is a key molecule in DNA double-stranded break repair [29]. This report suggested that an inhibitory small molecule of the FA/BRCA pathway could selectively kill tumor cells having other DNA repair protein deficiencies. Thus, FA/BRCA inhibitory chemicals might be used as cancer therapeutic tools in two ways. One is to resensitize tumor cells to ICL chemotherapy. The other is to achieve synthetic lethality with inhibition of other DNA repair-related proteins.

In an effort to identify novel anticancer therapeutics, we screened small molecules with inhibitory effects on FA/BRCA pathway activation upon ICL induction using FANCD2 nuclear foci as a quantitation marker for activation. Among the chemicals exhibiting inhibitory effects, ouabain was selected and its FA/BRCA pathway inhibiting activity was validated. Ouabain was confirmed to have sensitizing activity in U2OS cells to ICL-inducing agents at a concentration of 50 nM, which is just above IC_50_ (42.48 nM) for inhibition of FANCD2 activation (Figure 5A). Modifying the dose may make ouabain a good candidate for sensitization to conventional DNA ICL-inducing anticancer drugs including cisplatin. Supporting this notion, ouabain was reported to be a radiosensitizer in lung adenocarcinoma cells [30]. Furthermore, other cardiac glycosides such as digoxin and digitoxin showed synergism of cytotoxicity in combination with cisplatin in colon cancer cells [31].

Ouabain belongs to the cardiac glycoside family and is utilized in therapies for heart failure. However, the potential of ouabain as an anticancer agent was proposed in several recent publications. The cardiac glycoside family was reported to induce apoptosis and have an antiproliferative effect on various cancer cell lines (breast, prostate, melanoma, pancreatic, lung, leukemia, neuroblastoma, and renal adenocarcinoma) *in vitro* and *in vivo* [summarized in [19]]. Recently, digoxin, a cardiac glycoside, was identified as inhibiting prostate cancer cell proliferation *in vitro*, and its use is associated with a lower prostate cancer risk [32]. Through high-throughput drug repositioning screening, ouabain was identified to have anticancer activity against thyroid cancer cells [23]. UNBS 1450, a hemi-synthesized compound from cardiac glycosides, showed more competent antitumor activity than ouabain and digitoxin on non-small cell lung cancer in phase I clinical trials. Phase I clinical trials of Anvirzel, a cardiac glycoside, were recently conducted [19,33]. New applications for cardiac glycosides have been revealed in the cancer therapeutic field, but their exact mechanisms are not fully understood.

Cardiac glycosides affect several mechanisms. They trigger intracellular Ca^2+^ oscillations via Na^+^/K^+^ ATPase and inositol 1,4,5-triphosphate receptor (IP3R) binding [34]. In addition, cardiac glycoside binding to Na^+^/K^+^ ATPase activates the tyrosine kinase SRC-EGFR-Ras-Raf-MAPK signaling cascade [27]. These signal mechanisms mediate cell proliferation, differentiation, and apoptosis; increase ROS production; and alter gene expression profiles [19,20]. In this study, we suggest a new function of ouabain that inhibits the FA/BRCA pathway.

In previous reports, ouabain and cardiac glycosides could arrest cell cycle progression at G2/M phase in many tumor cells [23,24,35,36]. The mechanisms of cell cycle arrest by cardiac glycosides have not been revealed yet. In our study, ouabain did induce G2/M cell cycle arrest at 250 nM in U2OS cells consistent with the previous reports. At lower concentration (50 nM), ouabain did not affect cell cycle arrest, but did override MMC-induced cell cycle arrest (Figure 3). This effect of ouabain might potentiate cytotoxicity of MMC, but the underlying mechanism remains to be clarified.

The MAP kinase pathway was reported to react to the signal transduced from Na^+^/K^+^ ATPase [27]. Consistent with this, inhibition of p38 kinase with its specific inhibitor abolished FA/BRCA pathway inhibition by ouabain. This implies that p38 kinase activation may play an inhibitory role in FA/BRCA pathway activation. MAP kinases, JNK and p38, are activated when the FA/BRCA pathway is inhibited by gene silencing of FANCF in breast cancer cells [37]. A role for these kinases has been reported in hematopoiesis of FANCC-deficient cells [38]. However, the direct involvement of these kinases in the FA/BRCA pathway, to our knowledge, has not been reported. Further study regarding the mechanistic function of p38 kinase in FA/BRCA activation is warranted.

Previously, curcumin and menadione were reported to inhibit the FA/BRCA pathway [11,12]. In this study, ouabain was added to the list of chemicals with FA/BRCA pathway-inhibiting and chemosensitizing activities. Further mechanistic study of this activity of ouabain may lead to the development of chemosensitizers of ICL-inducing cancer therapeutics with wide clinical use.

## Supporting Information

Figure S1 **FANCD2 and FANCI transcriptional repression by ouabain in U2OS cell line.** (**A**) Ouabain reduced FANCD2 and FANCI expression in mRNA level. cDNAs from U2OS cells treated with the indicated concentration of ouabain or curcumin for 24 h were analyzed by real time PCR. Values represent the means ± SEM. (**B**) Inhibition of proteasomal degradation did not affect FANCD2 and FANCI protein expression. U2OS cells were treated with 100 nM ouabain or 5 µM curcumin for 24 h in the presence of MG132 or not. MG132 treatment was restricted to initial or later 4 h of ouabain-treating 24 h span.
(PDF)

Figure S2 **Effect of ouabain on ERCC4, FANCF, FANCA, RAD51, XRCC-5 and XRCC-6 mRNA expression in U2OS cell line.** cDNAs from U2OS cells treated with the indicated concentration of ouabain for 24 h were analyzed by real time PCR. Values represent the means ± SEM.
(PDF)

Figure S3 **FA-BRCA pathway inhibition by ouabain in HeLa cell line.** (**A**) Ouabain inhibits MMC-induced FANCD2 foci formation in HeLa cells. HeLa cells were pretreated with the indicated concentration of ouabain or curcumin for 1 h and then treated with 200 ng/ml MMC for 24 h. After incubation, the cells were fixed and processed for FANCD2 immunofluorescence and the FANCD2 foci were analyzed with an IN Cell Analyzer. Representative graphs and images from three independent experiments are shown. Values represent the means ± SEM. (**B**) Cardiac glycoside family members inhibit FANCD2 monoubiquitination. Protein extracts from HeLa cells, which were pretreated with 100 nM ouabain, 100 nM digitoxin, 100 nM digoxin and 20 µM curcumin and incubated in 200 ng/ml MMC for 24 h as described in (A), were analyzed by Western blotting using antibodies against FANCD2 and β-actin. L/S values shown represent the ratio of FANCD2 (L)/FANCD2 (S).
(PDF)

Figure S4 **Ouabain did not induce intracellular Ca2+ ion concentration fluctuation.** U2OS cells on chamber slide plate were treated with 1 µM fluo-4/2AM (Molecular Probe Inc.) + 0.02% pluronic F-127 (Invitrogen) in phenol red free DMEM for 30 min and then incubated in phenol red free DMEM for 30 min. After thapsigargin or ouabain were added to cells at indicated concentration, fluorescence intensity was determined every 20 sec for 20 min using confocal microscope.
(PDF)

Figure S5 **FA-BRCA pathway inhibition by ouabain is independent of intracellular Ca^2+^ ion increase.** U2OS cells were pre-treated with 10 µM nifedifine for 30 min and 50 nM ouabain for 30 min sequentially, and then incubated in 200 ng/ml MMC for 24 h. After incubation, the cells were fixed and processed for FANCD2 immunofluorescence, and the FANCD2 foci were analyzed with an IN Cell Analyzer. Representative graphs from three independent experiments are shown. Values represent the means ± SEM and MMC uptake regulation.
(PDF)

Figure S6 **FA-BRCA pathway inhibition by ouabain is not dependent on MMC uptake abrogation.** For pre-ouabain test, U2OS cells were pre-incubated with 100 nM ouabain for 1 h and incubated in medium containing 200 ng/ml MMC. For post-ouabain test, U2OS cells were incubated in medium containing 200 ng/ml MMC for 6 h. After incubation the cells were more incubation in MMC-free medium containing 100 nM ouabain or not for 18 h. After incubation, the cells were fixed and processed for FANCD2 immunofluorescence, and the FANCD2 foci were analyzed with an IN Cell Analyzer. Representative graphs and images from three independent experiments are shown. Values represent the means ± SEM.
(PDF)

Table S1 **List of selected chemicals that inhibit FA-BRCA pathway.**
(PDF)
