# Supplementary figures and images for: Ouabain, a Cardiac Glycoside, Inhibits the Fanconi Anemia/BRCA Pathway Activated by DNA Interstrand Cross-Linking Agents
Source: PLoS One. 2013 Oct 4;8(10):e75905. doi: 10.1371/journal.pone.0075905 (PMC3790830; doi:10.1371/journal.pone.0075905)

A

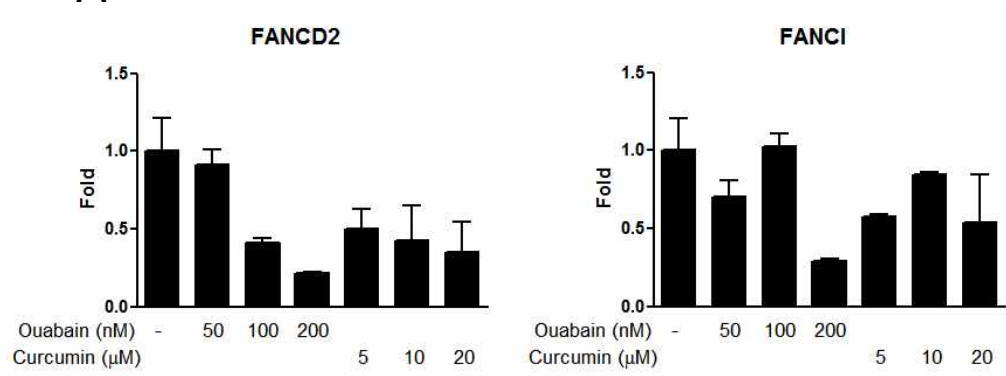

B

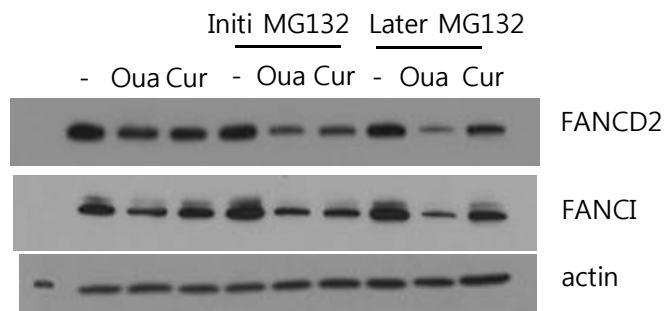

Supplement: Figure S1 — FANCD2 and FANCI transcriptional repression by ouabain in U2OS cell line. (A) Ouabain reduced FANCD2 and FANCI expression in mRNA level. cDNAs from U2OS cells treated with the indicated concentration of ouabain or curcumin for 24 h were analyzed by real time PCR. Values represent the means ± SEM. (B) Inhibition of proteasomal degradation did not affect FANCD2 and FANCI protein expression. U2OS cells were treated with 100 nM ouabain or 5 µM curcumin for 24 h in the presence of MG132 or not. MG132 treatment was restricted to initial or later 4 h of ouabain-treating 24 h span. (PDF) [file pone.0075905.s001.pdf]

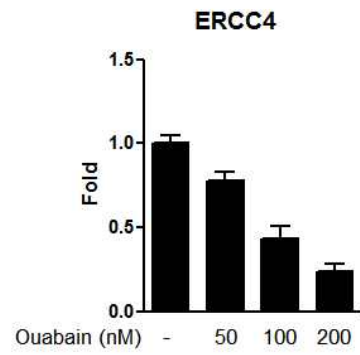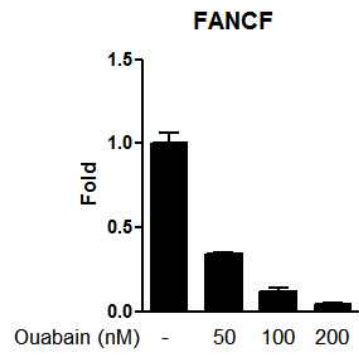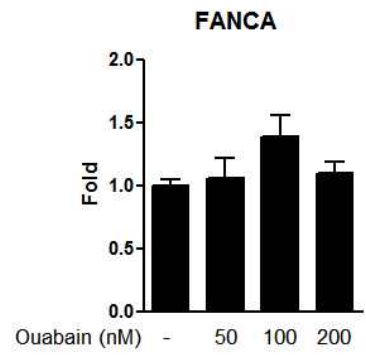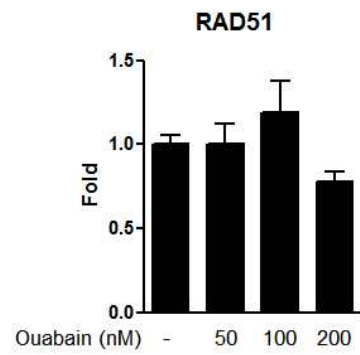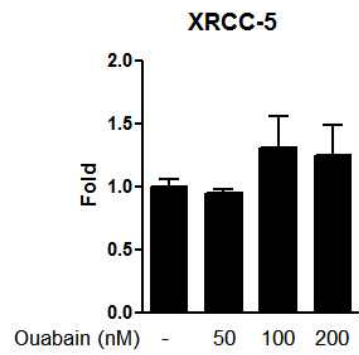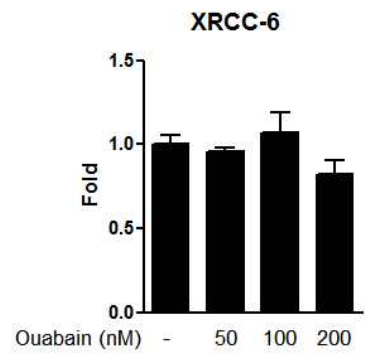

Supplement: Figure S2 — Effect of ouabain on ERCC4, FANCF, FANCA, RAD51, XRCC-5 and XRCC-6 mRNA expression in U2OS cell line. cDNAs from U2OS cells treated with the indicated concentration of ouabain for 24 h were analyzed by real time PCR. Values represent the means ± SEM. (PDF) [file pone.0075905.s002.pdf]

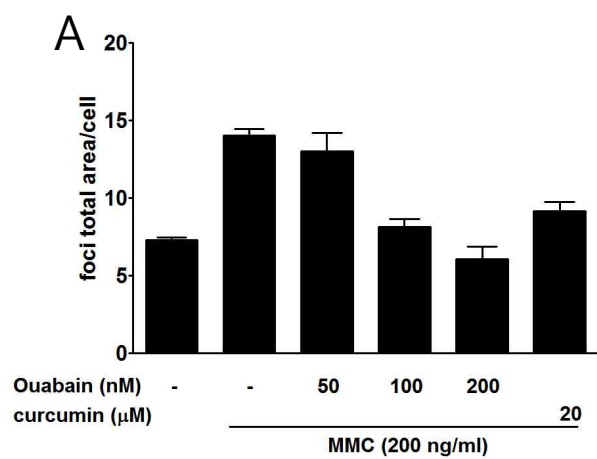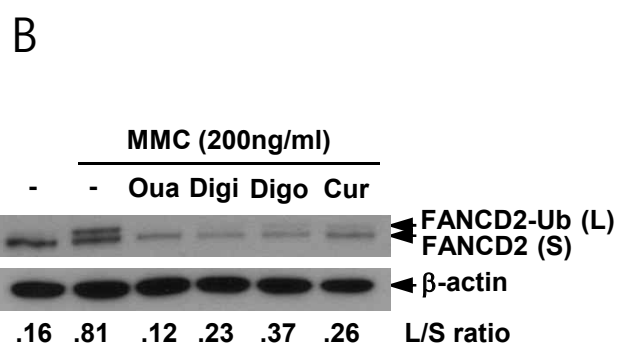

Supplement: Figure S3 — FA-BRCA pathway inhibition by ouabain in HeLa cell line. (A) Ouabain inhibits MMC-induced FANCD2 foci formation in HeLa cells. HeLa cells were pretreated with the indicated concentration of ouabain or curcumin for 1 h and then treated with 200 ng/ml MMC for 24 h. After incubation, the cells were fixed and processed for FANCD2 immunofluorescence and the FANCD2 foci were analyzed with an IN Cell Analyzer. Representative graphs and images from three independent experiments are shown. Values represent the means ± SEM. (B) Cardiac glycoside family members inhibit FANCD2 monoubiquitination. Protein extracts from HeLa cells, which were pretreated with 100 nM ouabain, 100 nM digitoxin, 100 nM digoxin and 20 µM curcumin and incubated in 200 ng/ml MMC for 24 h as described in (A), were analyzed by Western blotting using antibodies against FANCD2 and β-actin. L/S values shown represent the ratio of FANCD2 (L)/FANCD2 (S). (PDF) [file pone.0075905.s003.pdf]

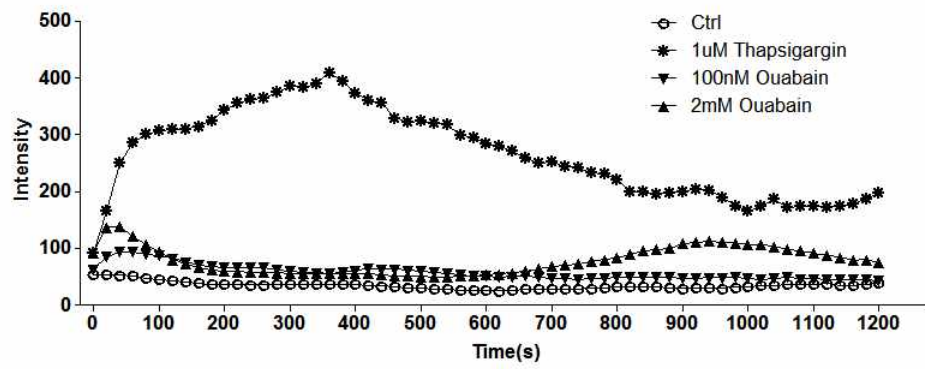

Supplement: Figure S4 — Ouabain did not induce intracellular Ca2+ ion concentration fluctuation. U2OS cells on chamber slide plate were treated with 1 µM fluo-4/2AM (Molecular Probe Inc.) + 0.02% pluronic F-127 (Invitrogen) in phenol red free DMEM for 30 min and then incubated in phenol red free DMEM for 30 min. After thapsigargin or ouabain were added to cells at indicated concentration, fluorescence intensity was determined every 20 sec for 20 min using confocal microscope. (PDF) [file pone.0075905.s004.pdf]

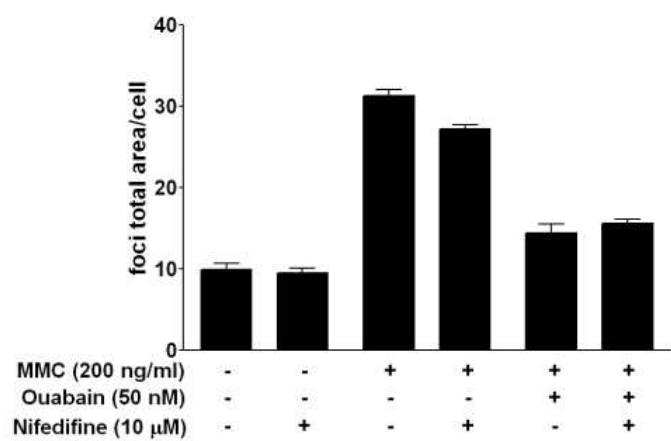

Supplement: Figure S5 — FA-BRCA pathway inhibition by ouabain is independent of intracellular Ca2+ ion increase. U2OS cells were pre-treated with 10 µM nifedifine for 30 min and 50 nM ouabain for 30 min sequentially, and then incubated in 200 ng/ml MMC for 24 h. After incubation, the cells were fixed and processed for FANCD2 immunofluorescence, and the FANCD2 foci were analyzed with an IN Cell Analyzer. Representative graphs from three independent experiments are shown. Values represent the means ± SEM and MMC uptake regulation. (PDF) [file pone.0075905.s005.pdf]

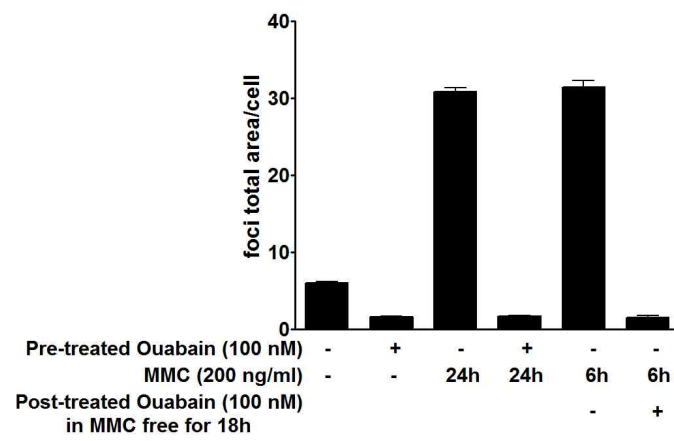

Supplement: Figure S6 — FA-BRCA pathway inhibition by ouabain is not dependent on MMC uptake abrogation. For pre-ouabain test, U2OS cells were pre-incubated with 100 nM ouabain for 1 h and incubated in medium containing 200 ng/ml MMC. For post-ouabain test, U2OS cells were incubated in medium containing 200 ng/ml MMC for 6 h. After incubation the cells were more incubation in MMC-free medium containing 100 nM ouabain or not for 18 h. After incubation, the cells were fixed and processed for FANCD2 immunofluorescence, and the FANCD2 foci were analyzed with an IN Cell Analyzer. Representative graphs and images from three independent experiments are shown. Values represent the means ± SEM. (PDF) [file pone.0075905.s006.pdf]
