# Supplementary material for: Ouabain, a Cardiac Glycoside, Inhibits the Fanconi Anemia/BRCA Pathway Activated by DNA Interstrand Cross-Linking Agents
Source: PLoS One. 2013 Oct 4;8(10):e75905. doi: 10.1371/journal.pone.0075905 (PMC3790830; doi:10.1371/journal.pone.0075905)
Supplement: Table S1 — List of selected chemicals that inhibit FA-BRCA pathway. (PDF) [file pone.0075905.s007.pdf]

Rank	Name	Description	FANCD2 foci area
1	Ouabain	Blocks movement of the H5 and H6 transmembrane domains of Na+-K+ ATPases	4.893
2	Dihydroouabain	Sodium-potassium pump inhibitor	6.266
3	Kenpaullone	Potent inhibitor of CDK1/cyclin B, CDK2/cyclin A, CDK2/cyclin E, and CDK5/p25	7.325
4	Cantharidic Acid	Protein phosphatase 1 (PP1) and 2A (PP2A) inhibitor	8.544
5	Cantharidin	Protein phosphatase 2A inhibitor	8.601
6	SU 9516	Cylcin-dependent kinase-2 (Cdk-2) inhibitor; induces apoptosis in colon cancer cells	10.064
7	CGP-74514A hydrochloride	Cdk1 inhibitor	11.023
